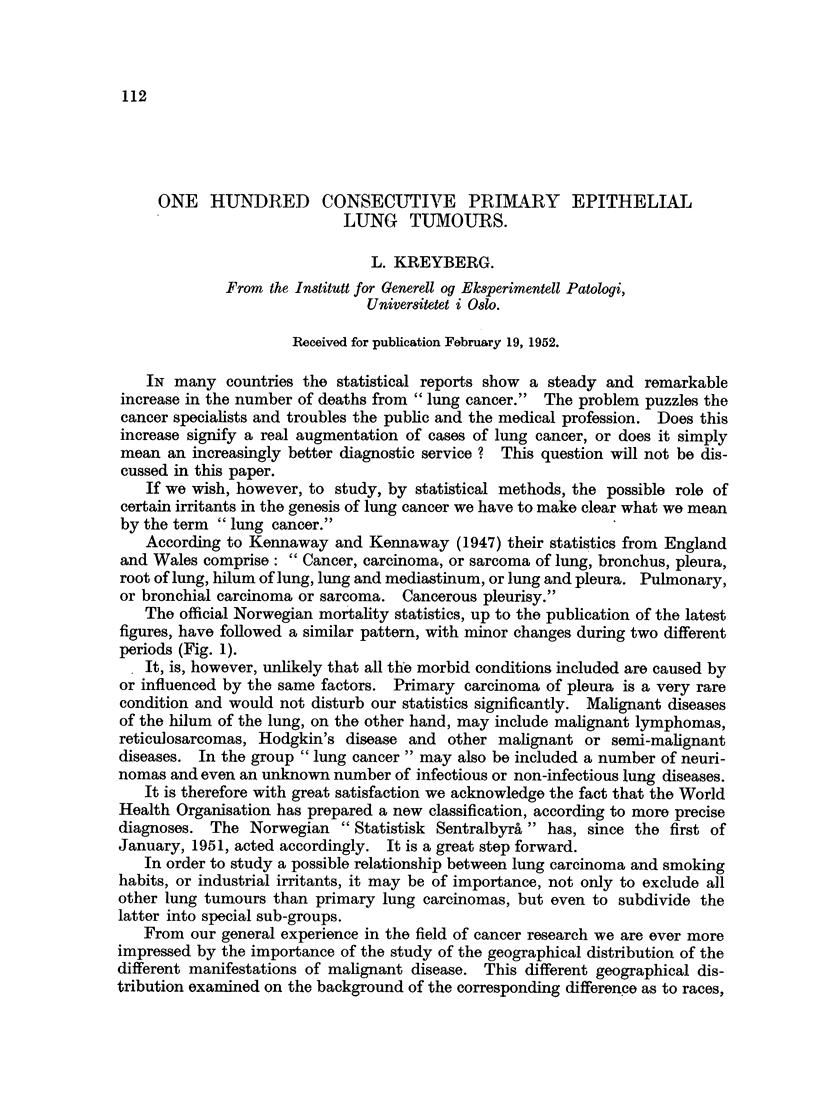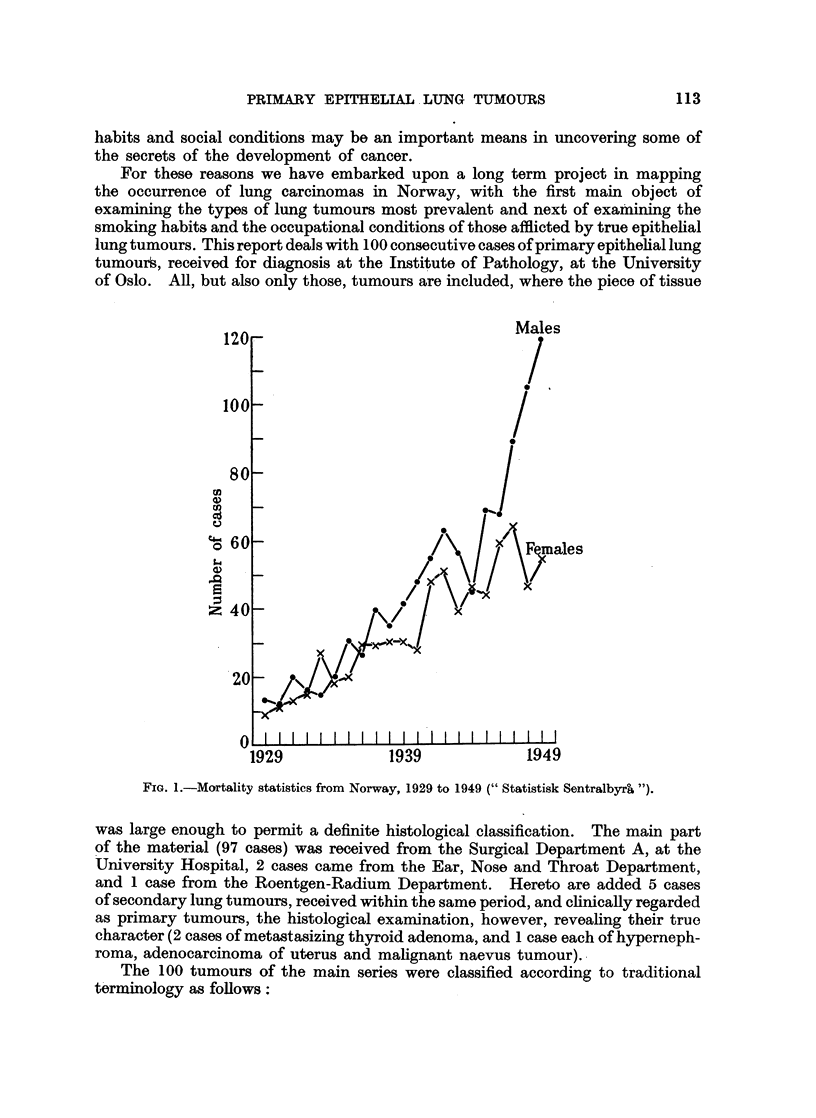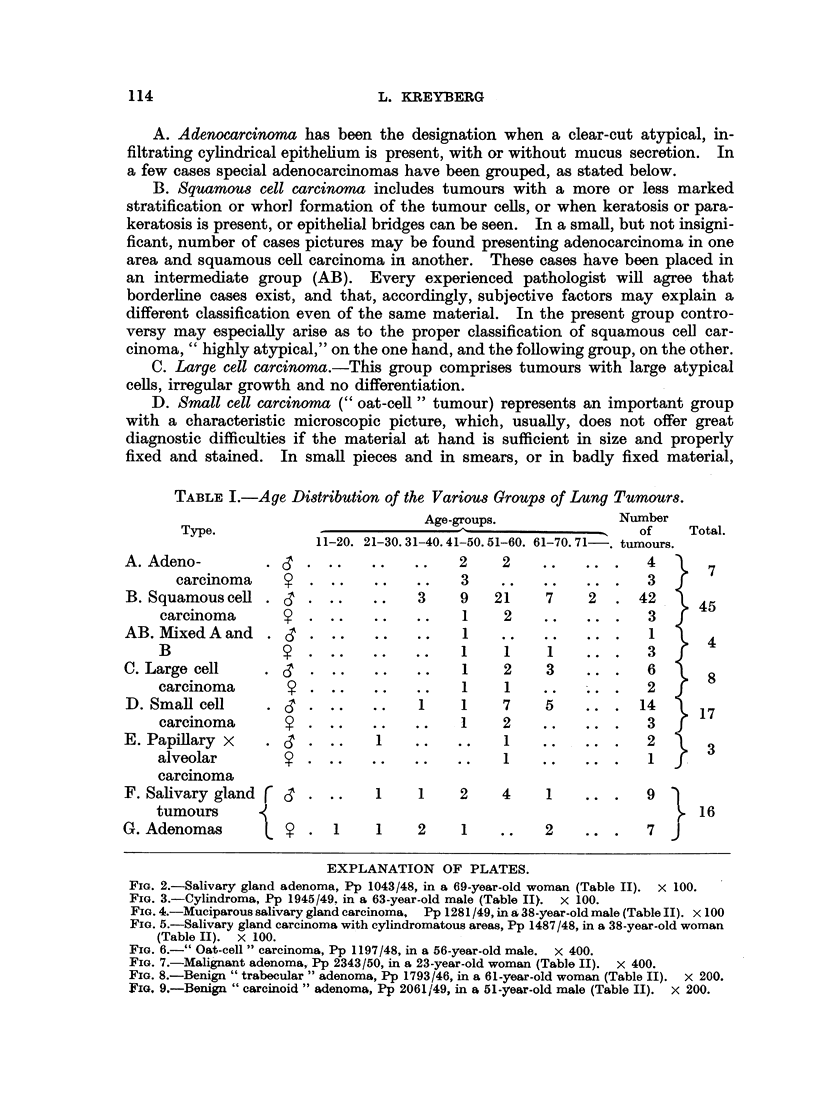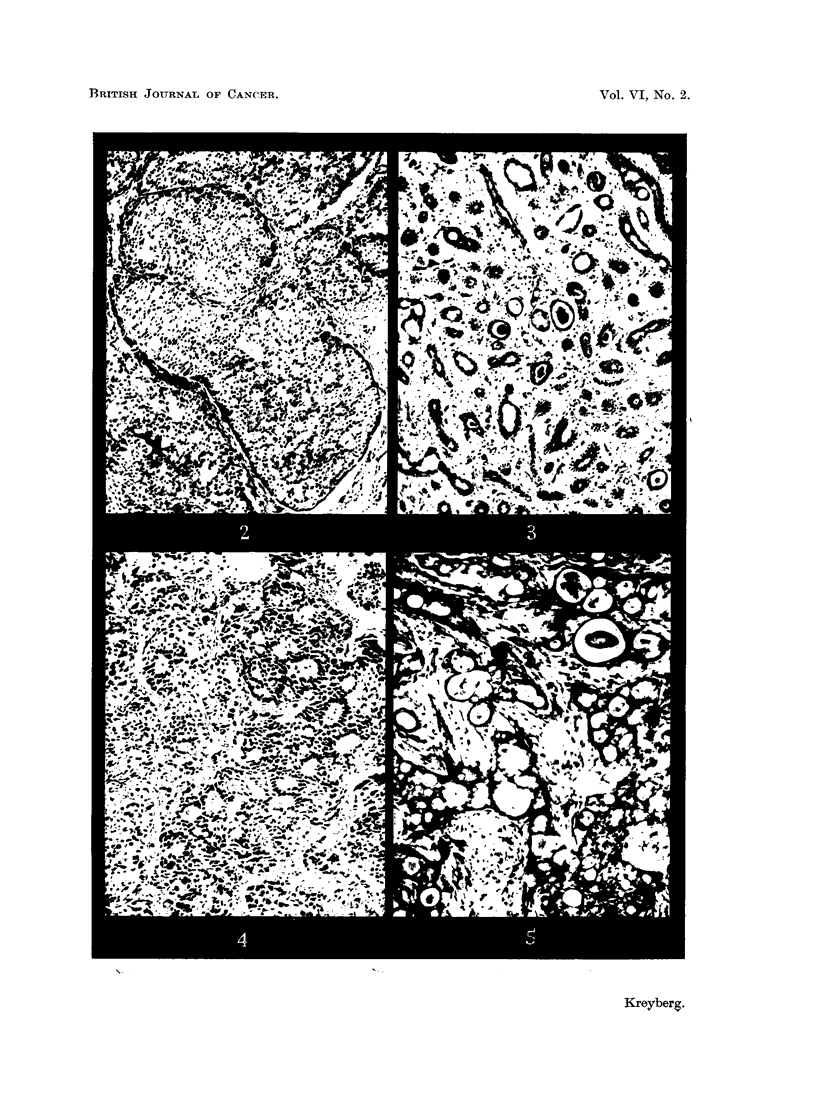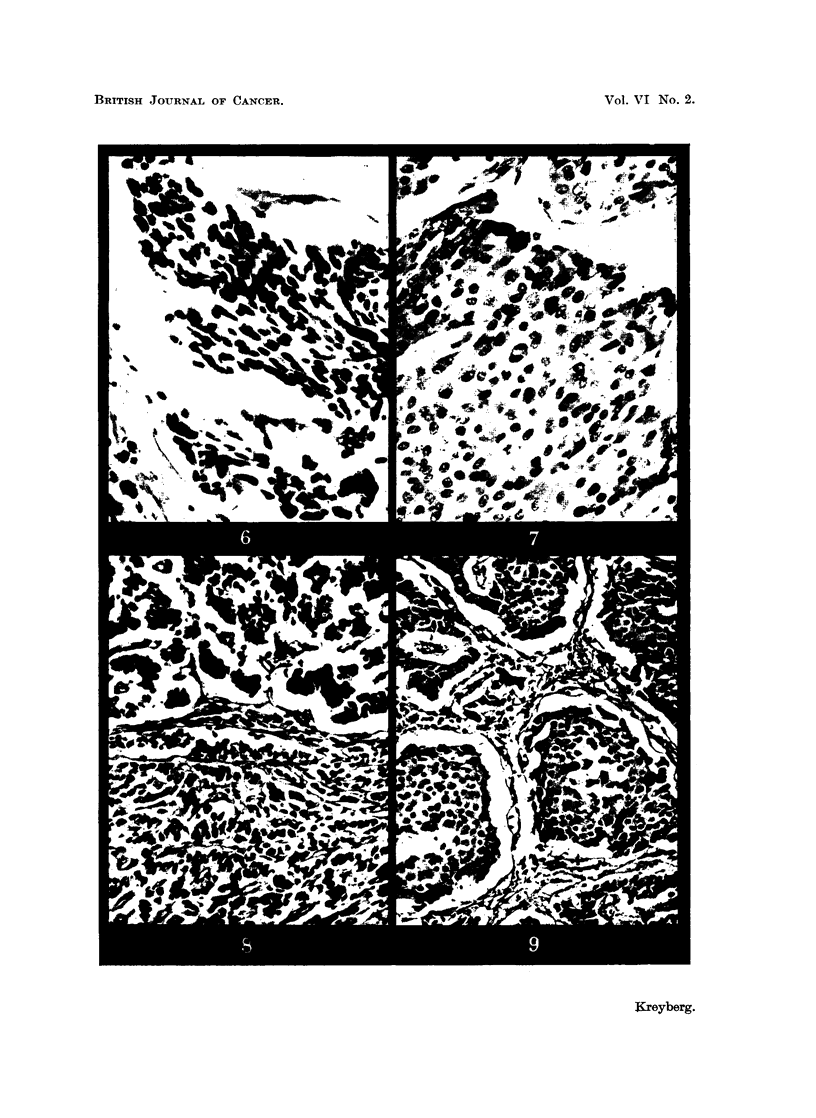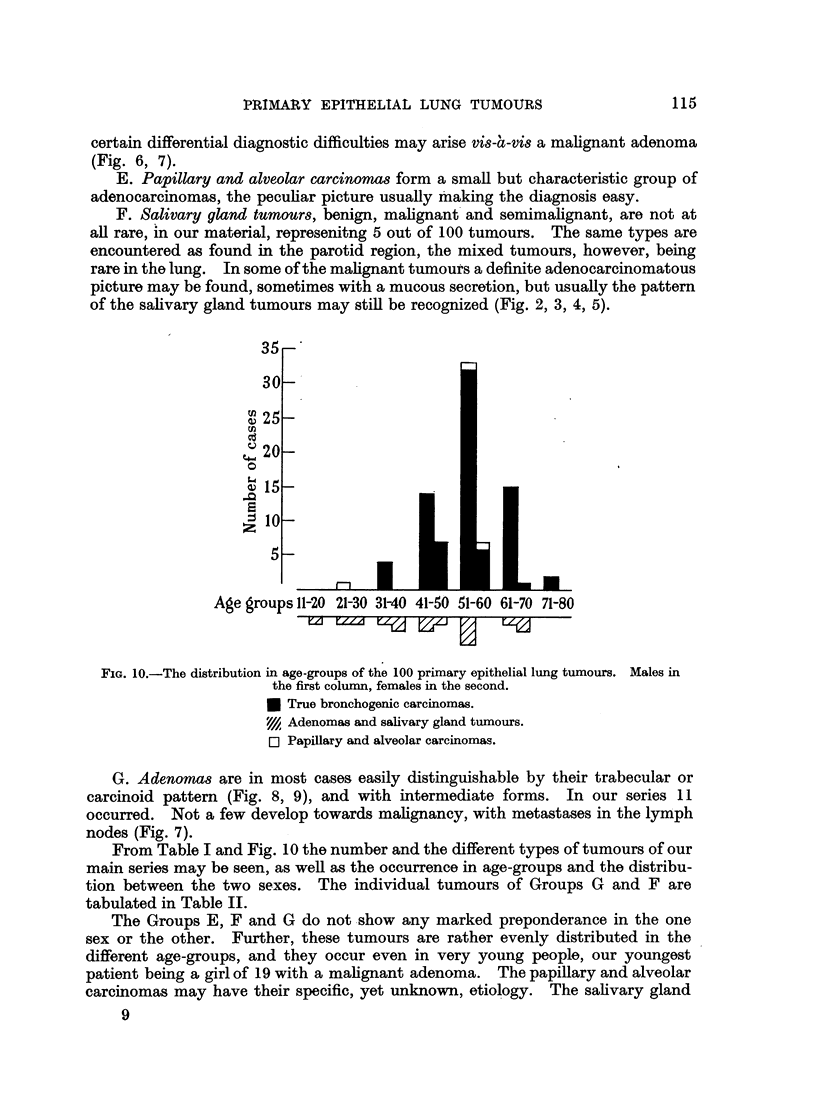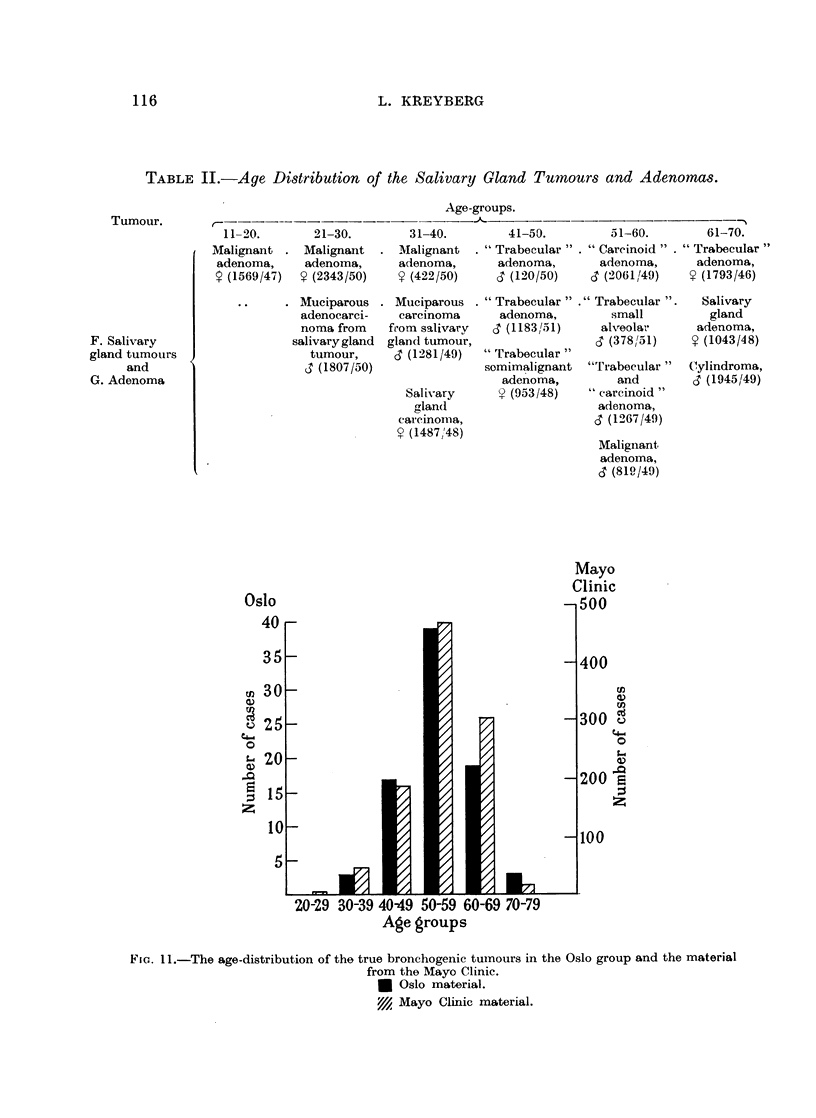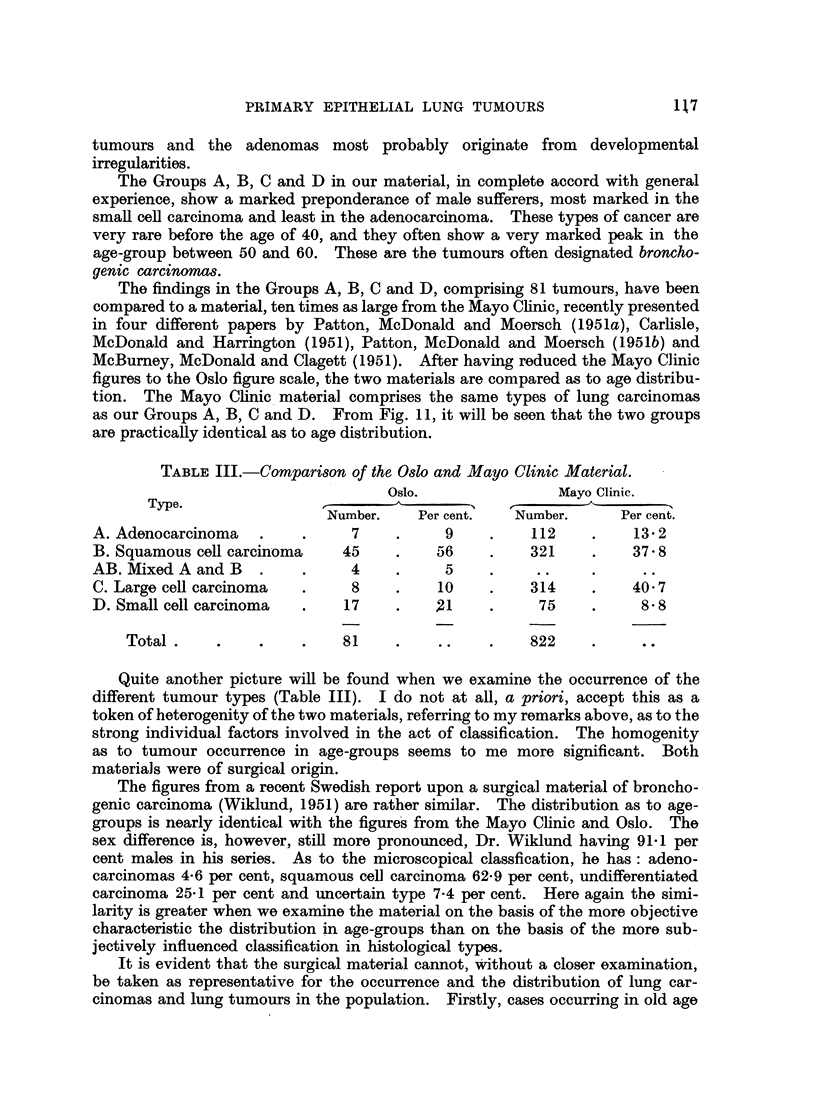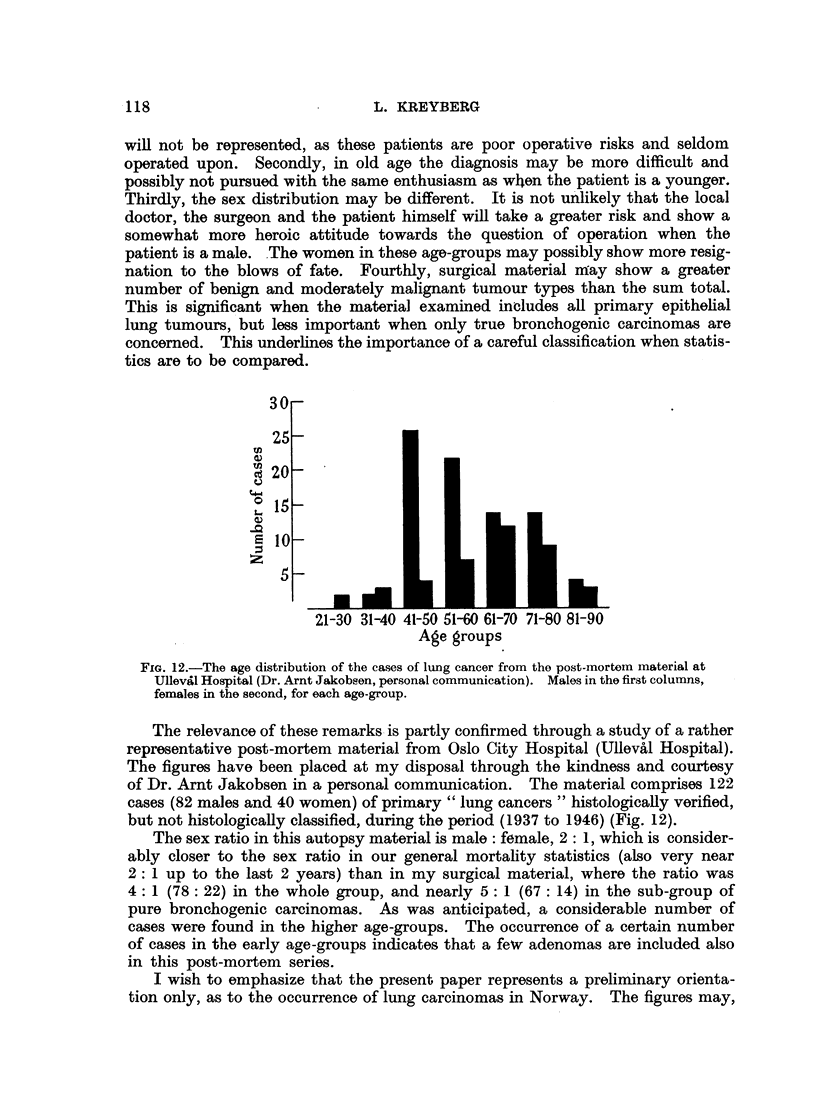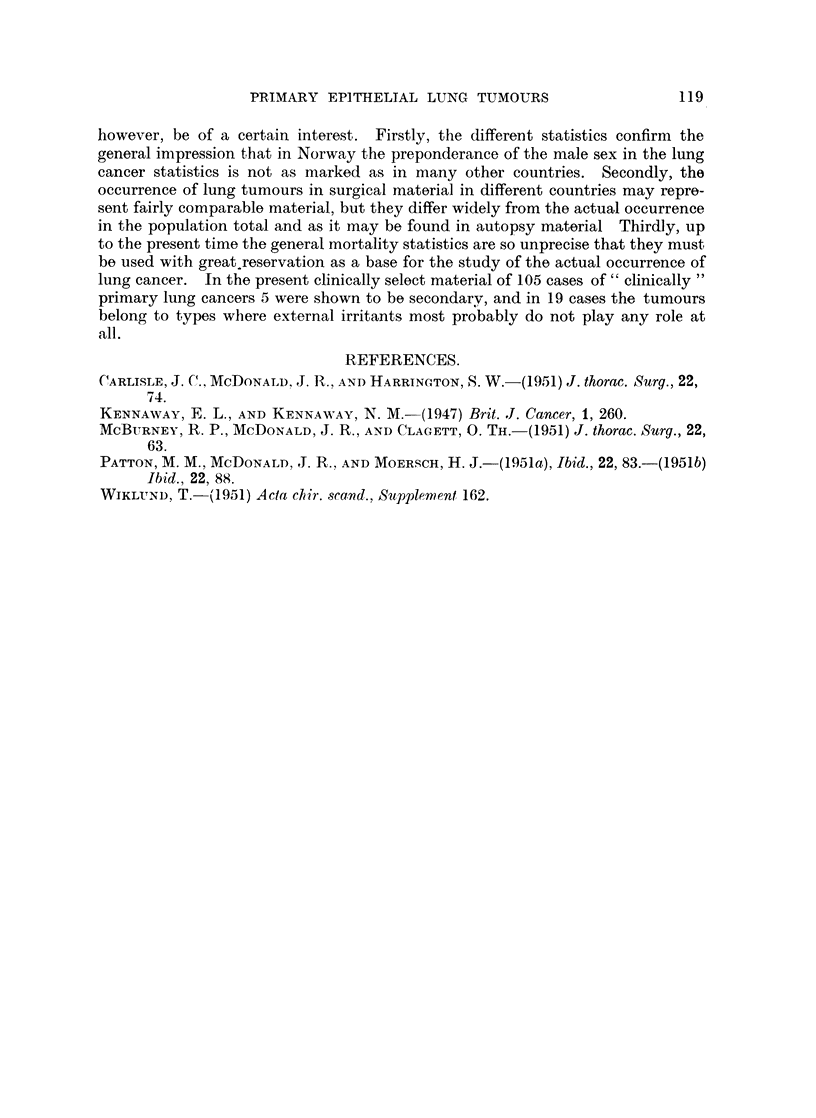# One Hundred Consecutive Primary Epithelial Lung Tumours

**DOI:** 10.1038/bjc.1952.12

**Published:** 1952-06

**Authors:** L. Kreyberg

## Abstract

**Images:**


### ONE HUNDRED CONSECUTIVE PRIMARY EPITHELIAL LUNG TUMOURS.

L. KREYBERG.

*From the Institutt for Generell og Eksperimentell Patologi, Universitetet i Oslo.*

Received for publication February 19, 1952.

In many countries the statistical reports show a steady and remarkable increase in the number of deaths from " lung cancer." The problem puzzles the cancer specialists and troubles the public and the medical profession. Does this increase signify a real augmentation of cases of lung cancer, or does it simply mean an increasingly better diagnostic service ? This question will not be discussed in this paper.

If we wish, however, to study, by statistical methods, the possible role of certain irritants in the genesis of lung cancer we have to make clear what we mean by the term " lung cancer."

According to Kennaway and Kennaway (1947) their statistics from England and Wales comprise : " Cancer, carcinoma, or sarcoma of lung, bronchus, pleura, root of lung, hilum of lung, lung and mediastinum, or lung and pleura. Pulmonary, or bronchial carcinoma or sarcoma. Cancerous pleurisy."

The official Norwegian mortality statistics, up to the publication of the latest figures, have followed a similar pattern, with minor changes during two different periods (Fig. 1).

It, is, however, unlikely that all the morbid conditions included are caused by or influenced by the same factors. Primary carcinoma of pleura is a very rare condition and would not disturb our statistics significantly. Malignant diseases of the hilum of the lung, on the other hand, may include malignant lymphomas, reticulosarcomas, Hodgkin's disease and other malignant or semi-malignant diseases. In the group " lung cancer " may also be included a number of neurinomas and even an unknown number of infectious or non-infectious lung diseases.

It is therefore with great satisfaction we acknowledge the fact that the World Health Organisation has prepared a new classification, according to more precise diagnoses. The Norwegian " Statistisk Sentralbyrå " has, since the first of January, 1951, acted accordingly. It is a great step forward.

In order to study a possible relationship between lung carcinoma and smoking habits, or industrial irritants, it may be of importance, not only to exclude all other lung tumours than primary lung carcinomas, but even to subdivide the latter into special sub-groups.

From our general experience in the field of cancer research we are ever more impressed by the importance of the study of the geographical distribution of the different manifestations of malignant disease. This different geographical distribution examined on the background of the corresponding difference as to races, habits and social conditions may be an important means in uncovering some of the secrets of the development of cancer.

For these reasons we have embarked upon a long term project in mapping the occurrence of lung carcinomas in Norway, with the first main object of examining the types of lung tumours most prevalent and next of examining the smoking habits and the occupational conditions of those afflicted by true epithelial lung tumours. This report deals with 100 consecutive cases of primary epithelial lung tumours, received for diagnosis at the Institute of Pathology, at the University of Oslo. All, but also only those, tumours are included, where the piece of tissue

FIG. 1.—Mortality statistics from Norway, 1929 to 1949 (" Statistisk Sentralbyrå ").

was large enough to permit a definite histological classification. The main part of the material (97 cases) was received from the Surgical Department A, at the University Hospital, 2 cases came from the Ear, Nose and Throat Department, and 1 case from the Roentgen-Radium Department. Hereto are added 5 cases of secondary lung tumours, received within the same period, and clinically regarded as primary tumours, the histological examination, however, revealing their true character (2 cases of metastasizing thyroid adenoma, and 1 case each of hypernephroma, adenocarcinoma of uterus and malignant naevus tumour).

The 100 tumours of the main series were classified according to traditional terminology as follows :

A. *Adenocarcinoma* has been the designation when a clear-cut atypical, infiltrating cylindrical epithelium is present, with or without mucus secretion. In a few cases special adenocarcinomas have been grouped, as stated below.

B. *Squamous cell carcinoma* includes tumours with a more or less marked stratification or whorl formation of the tumour cells, or when keratosis or parakeratosis is present, or epithelial bridges can be seen. In a small, but not insignificant, number of cases pictures may be found presenting adenocarcinoma in one area and squamous cell carcinoma in another. These cases have been placed in an intermediate group (AB). Every experienced pathologist will agree that borderline cases exist, and that, accordingly, subjective factors may explain a different classification even of the same material. In the present group controversy may especially arise as to the proper classification of squamous cell carcinoma, " highly atypical," on the one hand, and the following group, on the other.

C. *Large cell carcinoma.*—This group comprises tumours with large atypical cells, irregular growth and no differentiation.

D. *Small cell carcinoma* (" oat-cell " tumour) represents an important group with a characteristic microscopic picture, which, usually, does not offer great diagnostic difficulties if the material at hand is sufficient in size and properly fixed and stained. In small pieces and in smears, or in badly fixed material,

TABLE I.—*Age Distribution of the Various Groups of Lung Tumours.*

| Type. | | Age-groups. | | | | | | | Number of tumours. | Total. |
|---|---|---|---|---|---|---|---|---|---|---|
| | | 11–20. | 21–30. | 31–40. | 41–50. | 51–60. | 61–70. | 71—. | | |
| A. Adeno-carcinoma | ♂ | .. | .. | .. | 2 | 2 | .. | .. | 4 | 7 |
| | ♀ | .. | .. | .. | 3 | .. | .. | .. | 3 | |
| B. Squamous cell carcinoma | ♂ | .. | .. | 3 | 9 | 21 | 7 | 2 | 42 | 45 |
| | ♀ | .. | .. | .. | 1 | 2 | .. | .. | 3 | |
| AB. Mixed A and B | ♂ | .. | .. | .. | 1 | .. | .. | .. | 1 | 4 |
| | ♀ | .. | .. | .. | 1 | 1 | 1 | .. | 3 | |
| C. Large cell carcinoma | ♂ | .. | .. | .. | 1 | 2 | 3 | .. | 6 | 8 |
| | ♀ | .. | .. | .. | 1 | 1 | .. | .. | 2 | |
| D. Small cell carcinoma | ♂ | .. | .. | 1 | 1 | 7 | 5 | .. | 14 | 17 |
| | ♀ | .. | .. | .. | 1 | 2 | .. | .. | 3 | |
| E. Papillary × alveolar carcinoma | ♂ | .. | 1 | .. | .. | 1 | .. | .. | 2 | 3 |
| | ♀ | .. | .. | .. | .. | 1 | .. | .. | 1 | |
| F. Salivary gland tumours / G. Adenomas | ♂ | .. | 1 | 1 | 2 | 4 | 1 | .. | 9 | 16 |
| | ♀ | 1 | 1 | 2 | 1 | .. | 2 | .. | 7 | |

EXPLANATION OF PLATES.

FIG. 2.—Salivary gland adenoma, Pp 1043/48, in a 69-year-old woman (Table II). × 100.
FIG. 3.—Cylindroma, Pp 1945/49, in a 63-year-old male (Table II). × 100.
FIG. 4.—Muciparous salivary gland carcinoma, Pp 1281/49, in a 38-year-old male (Table II). × 100
FIG. 5.—Salivary gland carcinoma with cylindromatous areas, Pp 1487/48, in a 38-year-old woman (Table II). × 100.
FIG. 6.—" Oat-cell " carcinoma, Pp 1197/48, in a 56-year-old male. × 400.
FIG. 7.—Malignant adenoma, Pp 2343/50, in a 23-year-old woman (Table II). × 400.
FIG. 8.—Benign " trabecular " adenoma, Pp 1793/46, in a 61-year-old woman (Table II). × 200.
FIG. 9.—Benign " carcinoid " adenoma, Pp 2061/49, in a 51-year-old male (Table II). × 200.

Kreyberg.

Kreyberg.

certain differential diagnostic difficulties may arise *vis-à-vis* a malignant adenoma (Fig. 6, 7).

E. *Papillary and alveolar carcinomas* form a small but characteristic group of adenocarcinomas, the peculiar picture usually making the diagnosis easy.

F. *Salivary gland tumours*, benign, malignant and semimalignant, are not at all rare, in our material, represenitng 5 out of 100 tumours. The same types are encountered as found in the parotid region, the mixed tumours, however, being rare in the lung. In some of the malignant tumours a definite adenocarcinomatous picture may be found, sometimes with a mucous secretion, but usually the pattern of the salivary gland tumours may still be recognized (Fig. 2, 3, 4, 5).

FIG. 10.—The distribution in age-groups of the 100 primary epithelial lung tumours. Males in the first column, females in the second.

■ True bronchogenic carcinomas.

/// Adenomas and salivary gland tumours.

□ Papillary and alveolar carcinomas.

G. *Adenomas* are in most cases easily distinguishable by their trabecular or carcinoid pattern (Fig. 8, 9), and with intermediate forms. In our series 11 occurred. Not a few develop towards malignancy, with metastases in the lymph nodes (Fig. 7).

From Table I and Fig. 10 the number and the different types of tumours of our main series may be seen, as well as the occurrence in age-groups and the distribution between the two sexes. The individual tumours of Groups G and F are tabulated in Table II.

The Groups E, F and G do not show any marked preponderance in the one sex or the other. Further, these tumours are rather evenly distributed in the different age-groups, and they occur even in very young people, our youngest patient being a girl of 19 with a malignant adenoma. The papillary and alveolar carcinomas may have their specific, yet unknown, etiology. The salivary gland

TABLE II.—*Age Distribution of the Salivary Gland Tumours and Adenomas.*

| Tumour. | Age-groups. 11–20. | 21–30. | 31–40. | 41–50. | 51–60. | 61–70. |
|---|---|---|---|---|---|---|
| F. Salivary gland tumours and G. Adenoma | Malignant adenoma, ♀ (1569/47) | Malignant adenoma, ♀ (2343/50) | Malignant adenoma, ♀ (422/50) | " Trabecular " adenoma, ♂ (120/50) | " Carcinoid " adenoma, ♂ (2061/49) | " Trabecular " adenoma, ♀ (1793/46) |
| | .. | Muciparous adenocarcinoma from salivary gland tumour, ♂ (1807/50) | Muciparous carcinoma from salivary gland tumour, ♂ (1281/49) | " Trabecular " adenoma, ♂ (1183/51) | " Trabecular ". small alveolar ♂ (378/51) | Salivary gland adenoma, ♀ (1043/48) |
| | | | Salivary gland carcinoma, ♀ (1487/48) | " Trabecular " somimalignant adenoma, ♀ (953/48) | "Trabecular " and " carcinoid " adenoma, ♂ (1267/49) | Cylindroma, ♂ (1945/49) |
| | | | | | Malignant adenoma, ♂ (819/49) | |

FIG. 11.—The age-distribution of the true bronchogenic tumours in the Oslo group and the material from the Mayo Clinic.
■ Oslo material.
▨ Mayo Clinic material.

tumours and the adenomas most probably originate from developmental irregularities.

The Groups A, B, C and D in our material, in complete accord with general experience, show a marked preponderance of male sufferers, most marked in the small cell carcinoma and least in the adenocarcinoma. These types of cancer are very rare before the age of 40, and they often show a very marked peak in the age-group between 50 and 60. These are the tumours often designated *bronchogenic carcinomas.*

The findings in the Groups A, B, C and D, comprising 81 tumours, have been compared to a material, ten times as large from the Mayo Clinic, recently presented in four different papers by Patton, McDonald and Moersch (1951*a*), Carlisle, McDonald and Harrington (1951), Patton, McDonald and Moersch (1951*b*) and McBurney, McDonald and Clagett (1951). After having reduced the Mayo Clinic figures to the Oslo figure scale, the two materials are compared as to age distribution. The Mayo Clinic material comprises the same types of lung carcinomas as our Groups A, B, C and D. From Fig. 11, it will be seen that the two groups are practically identical as to age distribution.

TABLE III.—*Comparison of the Oslo and Mayo Clinic Material.*

| Type. | Oslo. | | Mayo Clinic. | |
|---|---|---|---|---|
| | Number. | Per cent. | Number. | Per cent. |
| A. Adenocarcinoma | 7 | 9 | 112 | 13·2 |
| B. Squamous cell carcinoma | 45 | 56 | 321 | 37·8 |
| AB. Mixed A and B | 4 | 5 | .. | .. |
| C. Large cell carcinoma | 8 | 10 | 314 | 40·7 |
| D. Small cell carcinoma | 17 | 21 | 75 | 8·8 |
| Total | 81 | .. | 822 | .. |

Quite another picture will be found when we examine the occurrence of the different tumour types (Table III). I do not at all, *a priori*, accept this as a token of heterogenity of the two materials, referring to my remarks above, as to the strong individual factors involved in the act of classification. The homogenity as to tumour occurrence in age-groups seems to me more significant. Both materials were of surgical origin.

The figures from a recent Swedish report upon a surgical material of bronchogenic carcinoma (Wiklund, 1951) are rather similar. The distribution as to age-groups is nearly identical with the figures from the Mayo Clinic and Oslo. The sex difference is, however, still more pronounced, Dr. Wiklund having 91·1 per cent males in his series. As to the microscopical classfication, he has : adenocarcinomas 4·6 per cent, squamous cell carcinoma 62·9 per cent, undifferentiated carcinoma 25·1 per cent and uncertain type 7·4 per cent. Here again the similarity is greater when we examine the material on the basis of the more objective characteristic the distribution in age-groups than on the basis of the more subjectively influenced classification in histological types.

It is evident that the surgical material cannot, without a closer examination, be taken as representative for the occurrence and the distribution of lung carcinomas and lung tumours in the population. Firstly, cases occurring in old age will not be represented, as these patients are poor operative risks and seldom operated upon. Secondly, in old age the diagnosis may be more difficult and possibly not pursued with the same enthusiasm as when the patient is a younger. Thirdly, the sex distribution may be different. It is not unlikely that the local doctor, the surgeon and the patient himself will take a greater risk and show a somewhat more heroic attitude towards the question of operation when the patient is a male. The women in these age-groups may possibly show more resignation to the blows of fate. Fourthly, surgical material may show a greater number of benign and moderately malignant tumour types than the sum total. This is significant when the material examined includes all primary epithelial lung tumours, but less important when only true bronchogenic carcinomas are concerned. This underlines the importance of a careful classification when statistics are to be compared.

FIG. 12.—The age distribution of the cases of lung cancer from the post-mortem material at Ullevål Hospital (Dr. Arnt Jakobsen, personal communication). Males in the first columns, females in the second, for each age-group.

The relevance of these remarks is partly confirmed through a study of a rather representative post-mortem material from Oslo City Hospital (Ullevål Hospital). The figures have been placed at my disposal through the kindness and courtesy of Dr. Arnt Jakobsen in a personal communication. The material comprises 122 cases (82 males and 40 women) of primary " lung cancers " histologically verified, but not histologically classified, during the period (1937 to 1946) (Fig. 12).

The sex ratio in this autopsy material is male : female, 2 : 1, which is considerably closer to the sex ratio in our general mortality statistics (also very near 2 : 1 up to the last 2 years) than in my surgical material, where the ratio was 4 : 1 (78 : 22) in the whole group, and nearly 5 : 1 (67 : 14) in the sub-group of pure bronchogenic carcinomas. As was anticipated, a considerable number of cases were found in the higher age-groups. The occurrence of a certain number of cases in the early age-groups indicates that a few adenomas are included also in this post-mortem series.

I wish to emphasize that the present paper represents a preliminary orientation only, as to the occurrence of lung carcinomas in Norway. The figures may, however, be of a certain interest. Firstly, the different statistics confirm the general impression that in Norway the preponderance of the male sex in the lung cancer statistics is not as marked as in many other countries. Secondly, the occurrence of lung tumours in surgical material in different countries may represent fairly comparable material, but they differ widely from the actual occurrence in the population total and as it may be found in autopsy material Thirdly, up to the present time the general mortality statistics are so unprecise that they must be used with great reservation as a base for the study of the actual occurrence of lung cancer. In the present clinically select material of 105 cases of " clinically " primary lung cancers 5 were shown to be secondary, and in 19 cases the tumours belong to types where external irritants most probably do not play any role at all.

### REFERENCES.

CARLISLE, J. C., MCDONALD, J. R., AND HARRINGTON, S. W.—(1951) *J. thorac. Surg.*, **22**, 74.

KENNAWAY, E. L., AND KENNAWAY, N. M.—(1947) *Brit. J. Cancer*, **1**, 260.

MCBURNEY, R. P., MCDONALD, J. R., AND CLAGETT, O. TH.—(1951) *J. thorac. Surg.*, **22**, 63.

PATTON, M. M., MCDONALD, J. R., AND MOERSCH, H. J.—(1951*a*), *Ibid.*, **22**, 83.—(1951*b*) *Ibid.*, **22**, 88.

WIKLUND, T.—(1951) *Acta chir. scand.*, *Supplement* 162.